# Space and Time Dynamics of Honeybee (*Apis mellifera* L.)-Melliferous Resource Interactions Within a Foraging Area: A Case Study in the Banja Luka Region (Bosnia & Herzegovina)

**DOI:** 10.3390/biology14040422

**Published:** 2025-04-15

**Authors:** Samuel Laboisse, Michel Vaillant, Clovis Cazenave, Biljana Kelečević, Iris Chevalier, Ludovic Andres

**Affiliations:** 1 Agricultural Development and Innovations for the Souths Research Unit (UR ADI-Suds), Engineering School in International Agro-Development (ISTOM), 49000 Angers, France; m.vaillant@istom.fr (M.V.); c.cazenave@istom.fr (C.C.); i.chevalier@istom.fr (I.C.); l.andres@istom.fr (L.A.); 2 Faculty of Agriculture, University of Banja Luka, 78000 Banja Luka, Bosnia and Herzegovina; biljana.kelecevic@agro.unibl.org

**Keywords:** *Apis mellifera* L., honeybee, honey production potential, landscape, land use, melliferous resources, pollen and nectar, seasonality, space and time variations

## Abstract

Interactions between honeybees and the environment are often difficult to achieve, particularly when the purpose is to optimize beekeeping production. The present study proposed to monitor the space-time variations of melliferous resources potentially exploited by colonies within a foraging area in Bosnia & Herzegovina, characterized by contrasting landscapes. The combination of methods involving Geographical Information Systems, floristic monitoring, and modelling enabled honey production potential to be calculated for the entire foraging area. In particular, the location of taxa, their abundance, diversity, and phenology enabled us to determine the spatial distribution and temporal variation of production potential. *Robinia pseudoacacia* and *Rubus* sp. made a major contribution. This potential was highly contrasted, with distant areas from the apiary more attractive than closer ones, depending on the moment. Specific periods, such as June were particularly conducive to establishing a high potential. Forest and grassland played a major role in the temporal succession, mainly because of the area covered, but moments with lower potential were supported by specific land uses (orchards). Land uses with a small surface area, such as orchards, wasteland, and riparian zones had a high potential per unit area, and improving the production potential within a foraging area could involve increasing these specific surfaces.

## Simple Summary

A better understanding of resource exploitation by honeybees is fundamental for beekeeping production. More specifically, the spatial and temporal variability of the exploration area around the hive helps beekeepers to anticipate production levels and quality. The present study aimed at understanding the fine variability of these resources in space and time in northern Bosnia & Herzegovina. The combined use of geographical information system tools, field observations, and modelling contributed to elaborate monitoring maps of potential honey production levels over time, and to assess the impact of different land uses on the area, particularly on the plant species that contributed most to this potential. Forest and grassland had better contribution on potential, respectively in May–June and in July–August. Nevertheless, some specific land uses contributed to increasing the honey production potential, such as orchards in late March to the beginning of April, and wasteland for some plant species.

## 1. Introduction

Animal pollination, at the forefront of which is entomophilic pollination, is of vital importance in the production of many fruits and seeds used in food and feed [1]. In this context, the foraging activity of honeybees *Apis mellifera* L. represents an interesting opportunity to optimize pollination of many crops, but also to ensure the reproductive function of plant biodiversity in natural environments. Among the diversity of pollinators that play a major role in pollination of ecosystems [2], the honeybee is singular because it is dependent not only on the beekeeper’s practices but also on its physiological and behavioral specificities [3]. Many publications deal with the loss of pollinators [4,5,6,7], but it seems crucial to understand the involvement of bees in the current landscape, the one that still allows us to survive [8].

Bees are eusocial insects. Each individual interacts with others within a colony, which is based in a hive in the case of beekeeping activity. This colony is spatially positioned within a territory, and interactions with landscape elements will determine its survival. Therefore, it is interesting to know in detail the spatial distribution of resources available among the plant diversity with the foraging area, which is defined as the area of interactions favoured by foragers to gather the various resources the colony needs to survive. In order to collect these resources (mainly nectar and pollen), they can explore the foraging area over quite long distances, in excess of ten kilometers [9]. Most often, however, these distances remain between 1 and 3 km in radius around the hive [10]. The variability of this distance depends on the type of habitat considered [9,11,12,13,14,15]. The structure of the landscape, particularly in terms of quality, quantity, density, homogeneity, and seasonal variations in availability, also has an influence on the definition of the foraging area for the colony in question [14]. Each type of landscape has a different performance, and even forests that are not considered a good type could have some interest according to the period or the specific honey that beekeeper wants to obtain [16].

The match between melliferous resources and bee foragers is at the heart of beekeepers’ strategies for optimizing the resources used by the colony and guiding bee management, particularly in the choice of location and/or productive activities [17,18]. Several studies have evaluated this spatial distribution, notably [19], which have modeled the dynamics of nectar supply within the landscape, and on several temporal scales, from very fine (3–4 days) to coarser (months). Therefore, the foraging area needs to be known in detail in order to give beekeepers the ability to evaluate a territory in terms of production potential. Nevertheless, the fine monitoring of space and time variations of melliferous resources available within a precise foraging area remains poorly evaluated in this context.

The melliferous resource can be considered in first approximation as a surface, for which the nectar and/or pollen supply is provided by a plant species [3]. However, except in special cases (intensive monocultures), several plant species coexist on a given surface, which can give rise to competition between different melliferous plants, as several plant species compete to provide the best possible resource for the bee, and hence to be visited. In such cases, we need to be able to reason about the bee’s choice of the most interesting melliferous resource.

A number of ethological characteristics influence the interest of a geographical position in terms of melliferous resources. Floral constancy is defined as the preference of foragers to visit only one resource at a time during a trip [20]. Many foragers are conditioned during their flight by the recruitment of scouts within the hive, with dancing and trophallaxing behaviors [21]. Numerous studies chose dance activity as a tool for assessing interaction with melliferous resources [14,22,23]. This choice allows the clear identification of resources actually foraged by the recruiters, even if some variability can exist between previous resources exploited by recruiters and the next resources visited by recruits [22]. On the other hand, it does not give access to the potential of the foraging area and hence to the possible anticipation of impacts of weather conditions or agricultural choices in cultivated areas.

Numerous studies have modelled the functioning of bee colonies, proposing dynamics in terms of population and foraging behavior (for example [24] for a review). The presence and attractiveness of sectors to pollinators as a function of the geographical characterization of the landscape have been investigated [25,26], but few of these studies also consider the ecological variation that may exist within these environments [27]. Geographic Information System (GIS) approaches are often used to characterize the landscape as part of a model of bee colony functioning. However, they are very often used for regional or national scales [3] which makes them difficult to apply to the assessment of melliferous resources around a hive within the foraging area. In order to model the space and time variations of honey production potential in fine detail within the restricted territory of the foraging area, the present work proposes an approach combining a geographical approach using GIS tools, botanical inventories, phenological monitoring of flowering, and mathematical modelling. By taking into account not only the landscape but also plant development, we can gain a more accurate picture of variations in the nutritional resources available to colonies.

## 2. Materials and Methods

### 2.1. Study Site

**Figure 1:**
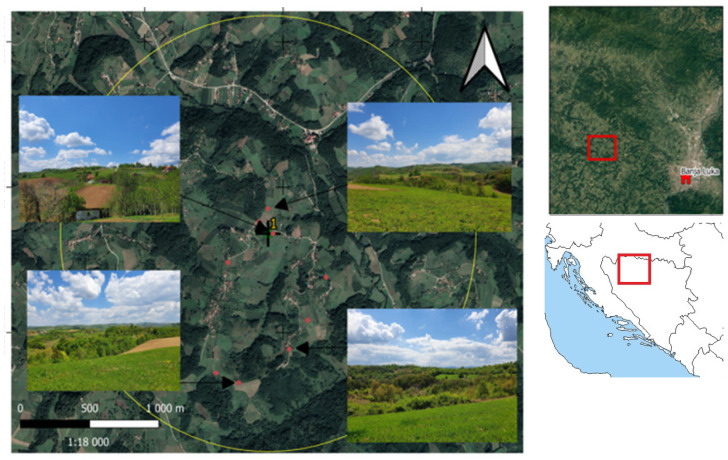
Location of the area and examples of landscapes. The study area is located in northern B&H, North-West of Banja Luka.

Bosnia and Herzegovina (B&H) is located in the western part of the Balkans Peninsula. The north comprises the southern edge of the Pannonian plain, belonging to the peripannonian region. The study area was located near the village of Bistrica (44.81° N, 17.05° E), on the territory of the region of Banja Luka, The Republic of Srpska (B&H). As suggested by [14], a radius of 1.5 km around the apiary location was used to define the foraging area. This territory is characterized by a heterogeneous landscape and a relief structuring the landscape into valleys and hills, forests, field crops, orchards, and residential areas that follow one another without really dominating the landscape (Figure 1). Landscape observation and preliminary GIS assessment were carried out at the start of the study to confirm the heterogeneous nature of the area.

### 2.2. Environmental Data

Field monitoring was carried out over two consecutive years (2022 and 2023) to capture inter-annual variability. These years were consistent in terms of average, minimum, and maximum temperatures, with the typical trend for this area (Table 1).

### 2.3. Characterisation of Land Use Using a Combined Field and GIS Approach

All geospatial analyses were represented with QGIS software, version 3.22.15 [31]. The aim was to create a graphical representation that shows the Land use Station (LUS) [32]. This is a spatial entity considered homogeneous, with specific biophysical characteristics that it covers (without taking into account its socio-economic use, although the two are linked). Therefore, it corresponds to the whole surface with the same land use, although it is not necessarily continuous. In order to refer more specifically to a continuous area with the same land use, we defined the term Land Use Unit (LUU). This is the spatial structuring unit of the foraging area in question. Two databases were used in the foraging area. The first was the “WorldCover 10 m 2020” developed by [33]. With a resolution of 10 m, this image is derived from remote sensing data from the Sentinel-1 and Sentinel-2 satellites. The second used the NDVI (Normalized Difference Vegetation Index) to highlight differences in vegetation density over the foraging area under consideration [34]. It was calculated using the 4 Sentinel-2 L2A red and 8 Sentinel-2 LSA near infrared spectral bands [33].

In addition to these GIS approaches, further field observations were carried out in order to refine the initial representations of land use. For all of the forest sectors, main tree species were evaluated along transects, and LUU was divided up if changed. All the crop fields were observed on site to identify orchards and plots of arable land. For this land use, only wheat, barley, and maize plots were identified. Maize was isolated because can play a role in bee nutrition as a pollen source. For most field crops, crop rotation was very low or non-existent in the study area. Therefore, we decided to fix the land use of field crops for the year 2023. For wasteland, the representation based on satellite images was not very accurate. It was identified by analysing aerial photographs using Google Earth software version 10.78.0.2 [35] and verifying them in the field (geolocated field surveys using the Field Maps application developed by ArcGIS Pro [36]). Riparian vegetation was very difficult to characterize by using GIS tools alone, and therefore it is very rarely mentioned in similar research [32]. Streams were exhaustively mapped in the foraging area during the month of April, after snowmelt and heavy rainfall, to increase their visibility, including temporary ones [37]. Each stream was followed from downstream to upstream and all the bends and branches encountered were recorded using Field Maps, in order to map the structure of the associated riparian forest in detail. In this way, eight LUSs were selected and described: forest, grassland, wasteland, riparian forest, arable crops (excluding maize), maize crops, orchards, habitations and others.

### 2.4. Analysis of Landscape Structure

Two levels of scale are used to assess the overall organization landscape within the foraging area under consideration: the LUU, which characterises the degree of fragmentation within the area, and the general landscape, which characterises its diversity [38]. For a quantitative approach, various indicators from the Fragstats software version 4.2 [39] were used, as detailed below.

$S_{i}$ is the average surface area of LUU for a given LUS i (ha), which gives an idea of the average size of LUU within an area:

(1) $$S_{i}=\frac{\sum_{j=1}^{n_{i}}a_{ij}}{n_{i}}$$

With $a_{ij}$ the surface area (m^2^) of the LUU ij and $n_{i}$ the total number of LUU within a LUS i.

${Dt}_{i}$ is the density of LUU per 100 ha for a LUS i. This gives an idea of the fragmentation of LUU and hence of the landscape:

(2) $${Dt}_{i}=\frac{n_{i}}{A}\left(10,000\right)(100)$$

where A is the total area of the landscape (m^2^).

$ENN_{i}$ is the average distance between two LUU of the same LUS i (m), which makes it possible to estimate the connectivity and dispersion of land use types:

(3) $$ENN_{i}=\frac{\sum_{j=1}^{n_{i}}d_{\mathrm{ij}}}{n_{i}}$$

where $d_{\mathrm{ij}}$ is the distance between two nearest neighboring spots of the same LUS i (m).

### 2.5. Characterisation of Melliferous Flora

#### 2.5.1. Sampling Method

In 2022, systematic sampling was set up to cover the entire foraging area. 150 m stretch separated each observation point from its neighbors in all directions. Only points that were inaccessible in the field or were not in exploitable areas were not determined. A total of 142 points were studied. The year 2023 was used to take account of inter-annual changes and to provide a more detailed description of certain areas/landscapes that are often given little consideration in this type of study. The observation points were placed according to a stratified sampling strategy. Each type of land use was assessed by setting up botanical observation points, with the exception of inhabited areas which were too difficult to access and fields cultivated with grasses, which are all characterized by the very low percentage of weeds present, the absence of rotations involving melliferous crops and the low melliferous interest of the main crops [40]. Within each type, the number of observation points was determined based on the share of the surface area allocated to the foraging area and the botanical diversity characterized in 2022. Each observation point was positioned at random within the LUUs of the same type.

#### 2.5.2. Botanical Parameter

At each observation point, a circular plot was positioned, the area of which depended on the stratum considered [41]. For an herbaceous layer (height less than 0.5 m) the radius was 0.56 m. For a shrub layer (height between 0.5 and 2 m) the radius was 4 m. Finally, for a tree layer (height greater than 2 m), the radius was 8 m. Within this plot, all the species were taken into account. Only grasses were not considered. The nomenclature and determination of species were conducted using modern systematic principles according to [42,43], and specific references linked to the region [44,45,46,47,48].

#### 2.5.3. Phenological Parameter

The floral development of the various melliferous species was monitored. Three specific phenophases, which can have an impact on nectar availability for bees, were considered on the basis of [49]:

- the early flowering phase, where less than 50% of the flowers were open (the rest were in the form of flower buds),
- the full flowering phase, where more than 50% of the flowers were open,
- the late flowering phase, where less than 50% of the flowers were open and the rest were wilted.

A qualitative assessment by direct observation for each melliferous species present at the observation points was carried out over weeks 10 to 34, from March to August to describe the evolution of phenology.

#### 2.5.4. Ecological Parameter

For each taxon present within an LUU, the rate of coverage was assessed using the Braun-Blanquet abundance-dominance coefficient methodology [50,51]. The total plant species, which is the total number of species present on a LUS, and the average plant species number per plot for each LUS, defined as the quotient of the sum of the species and the number of LUU for the LUS in question were evaluated.

### 2.6. Modelling

#### 2.6.1. Estimation of Honey Production Potential

Initially developed by [52] and taken up by [32], the honey production potential (HPP), which includes the main parameters that affect the attractiveness of a melliferous resource [53], was calculated by integrating the various parameters developed above, in particular phenology, in order to accurately monitor the temporal variability of the resources. This landscape element represents the sum of the HPPs of each melliferous species present in the LUU under consideration. It varied on a weekly basis, in contrast to previous studies:

(4) $$HPP=\left(1-\frac{D}{10,000}\right)*S*\sum_{t}(R_{t}*{pnh}_{t})$$

With:

HPP the honey production potential per LUU and per unit of time (kg/week),

t the number of considered nectariferous taxons,

D the distance between the center of gravity of the LUU and the apiary (m),

S the area covered by the LUU (ha),

$R_{t}$ the coverage rate of the taxon on the LUU worked (%),

${pnh}_{t}$ the weekly nectariferous potential of the taxon t (kg/ha), calculated as follows:

(5) $${pnh}_{t}=\frac{{pn}_{t}}{Sf}I_{f}$$

With:

${pn}_{t}$ the annual nectariferous potential of a given taxon t. This value was taken from the literature, in particular [46,47,48,52,54,55]. For species not listed in the literature, either values for the generic genus were still used if available (for *Campanula* sp., *Knautia* sp., *Ranunculus* sp., *Rubus* sp., *Salix* sp. and *Stachys* sp.), or they were not included due to lack of data,

Sf the number of weeks of flowering,

$I_{f}$ the flowering intensity, value 0.5 for early and late flowering, 1 for full flowering, 0 otherwise.

#### 2.6.2. Mapping the Space and Time Evolution of Melliferous Resources

For each LUU considered, the botanical, phenological, and ecological parameters used to calculate an HPP were associated with at least one observation made within the LUU. For the others, the nearest observation point located on another LUU was used to feed the equation. In this way, each pixel making up the foraging area was characterized by an LUU identification and a HPP value per week. This information was then vectorized on the mapping of the area using QGIS software in order to visualise the space and time variability of the HPP over the entire foraging area.

### 2.7. Statistical Analysis

A non-parametric test (Kruskal-Wallis test) and a two-by-two multiple comparison test were carried out to assess the significance of the effect of the LUS on the mean LUU surface area, the mean distance between the nearest LUUs and the average plant species number per observation plot, using R version 4.0.4 [56].

## 3. Results

### 3.1. Landscape Characterisation

**Figure 2:**
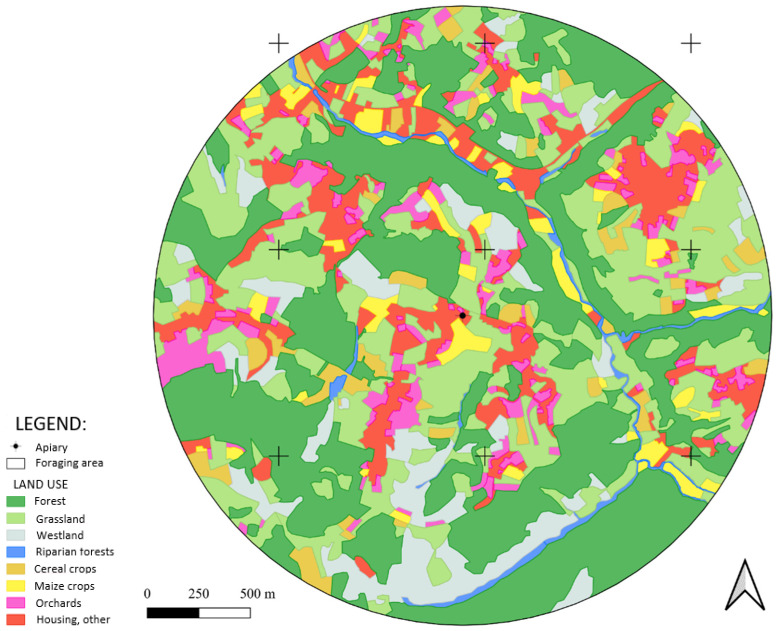
Distribution of land use types within the foraging area.

The total surface area of the foraging area was 707 ha. 36.3% was covered by forest, 26.1% by grassland, 12.5% by housing, 9.06% by wasteland, 6.11% by orchards, 4.17% by arable crops excluding maize, 4.04% by maize and 1.71% by riparian vegetation (Figure 2). The average surface area of the LUU was fairly small (1.75 ha), with cultivated LUU being even smaller on average, at around 0.5 ha (Table 2). Only the forested LUUs were larger, although they vary greatly in size. The landscape mosaic was particularly represented by the grassland and orchard LUU, which have a higher ${Dt}_{i}$ and a lower $ENN_{i}$ than the other LUSs. On the other hand, riparian vegetation was very little represented in the landscape and was exclusively linked to water (therefore very punctual in the landscape and linked to the watercourse and the small lakes present in the foraging area). The arable LUUs were particularly remote from one another. It should be noted that the average distance between two LUUs of the same type varies greatly from one LUS to another.

### 3.2. Link Between Land Use and Botanical Diversity

Total plant species were highest in grassland throughout the foraging area (Table 3). Orchards and wasteland had the lowest one. For this first LUS, non-cultivated flora was considered a weed and the management contributed to limiting the associated diversity. In the case of wasteland, these areas were in transition and were mainly colonized by plants considered to be pioneers or even ruderals, with fairly strong competition, particularly from *Rubus* sp. On the other hand, if we considered the number of species present on average within a plot, the diversity was greater for grassland and riparian zones, unlike for woodland and wasteland. Thus, the use of a restricted area (a LUU) by bees would allow a greater potential number of species to be used. The average plant species number for the orchard plots had not been evaluated, as the measurement process was different for this LUU, as most of the plots were inaccessible. An average value based on the only accessible plots was therefore generalized to the entire LUS.

### 3.3. Spatial and Temporal Distribution of HPP

**Figure 3:**
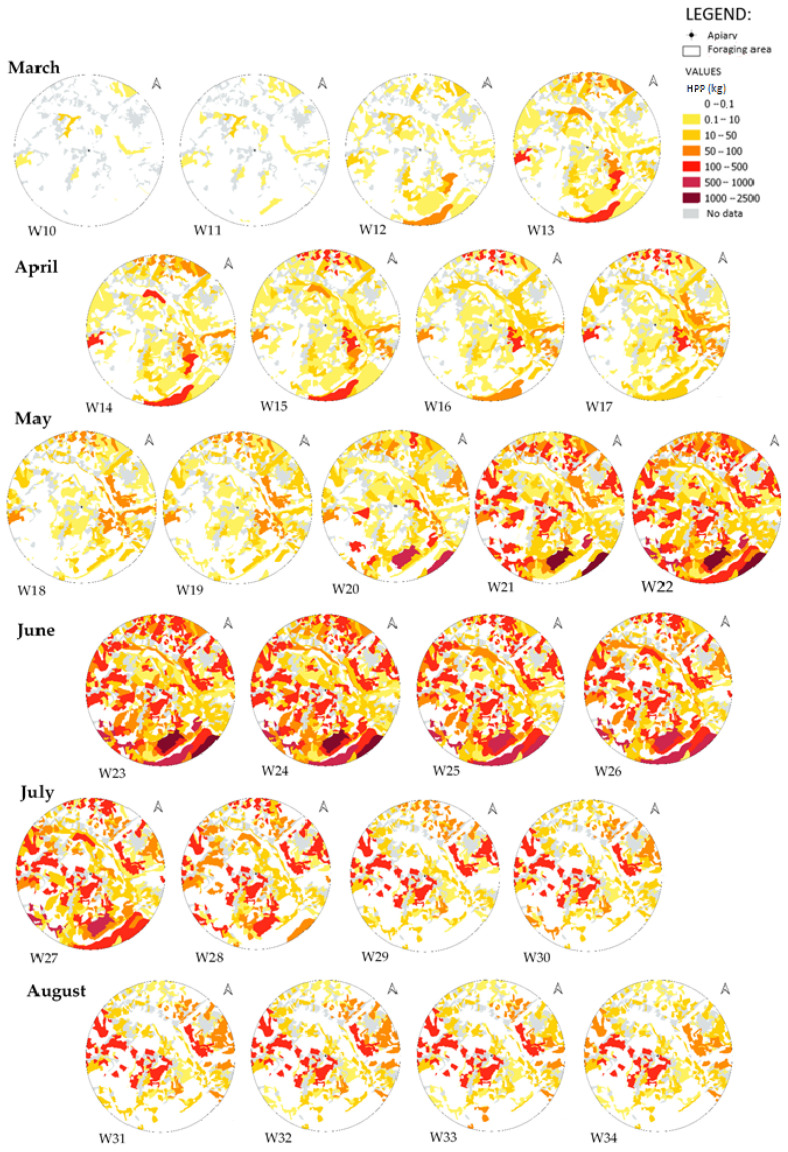
Space and time variation of HPP. The represented time step is the week, each card is linked to a week number during the year (for example W10 for week 10). Each circle has a diameter equivalent to 3 km.

The detailed space and time characterization of the foraging area (Figure 3) enabled the HPP to be monitored precisely over the weeks making up the beekeeping season. From a very low potential in the first few weeks (W10 and W11), concentrated on a few LUUs giving access to HPP of a few dozen kg but relatively far from the apiary, to a more substantial potential in W12 and W13, this led to a general increase in HPP in the area (W14 to W20), where less than half of the LUUs exceed an HPP of 10 kg and some were already at 100 kg or more, culminating in the most generous periods between W21 and W27, where almost all the LUUs were likely to provide an HPP of more than 50 kg. From W28 onwards, there were specific areas that were very attractive among others less generous. These areas of interest remained relatively close to the apiary.

### 3.4. Temporal Variations in Cumulative Weekly HPP in the Foraging Area

**Figure 4:**
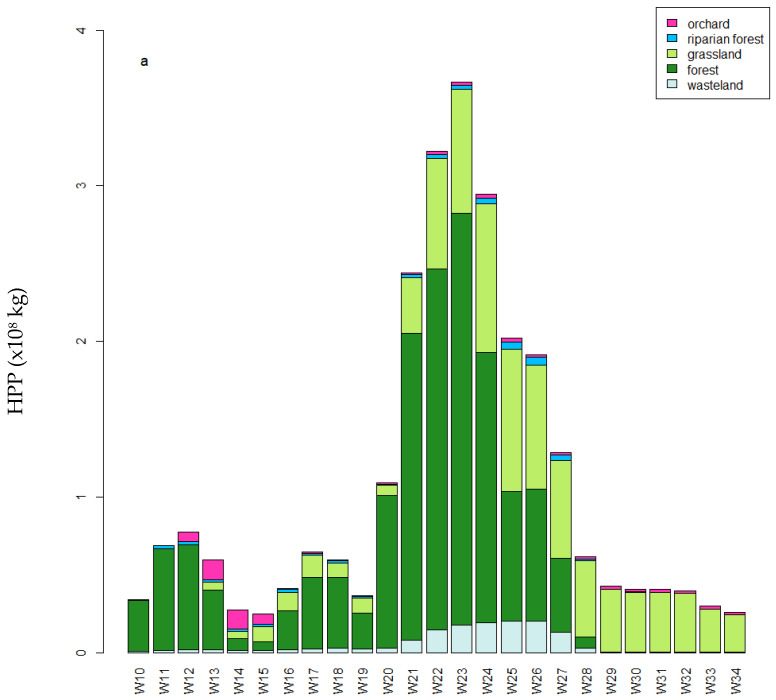

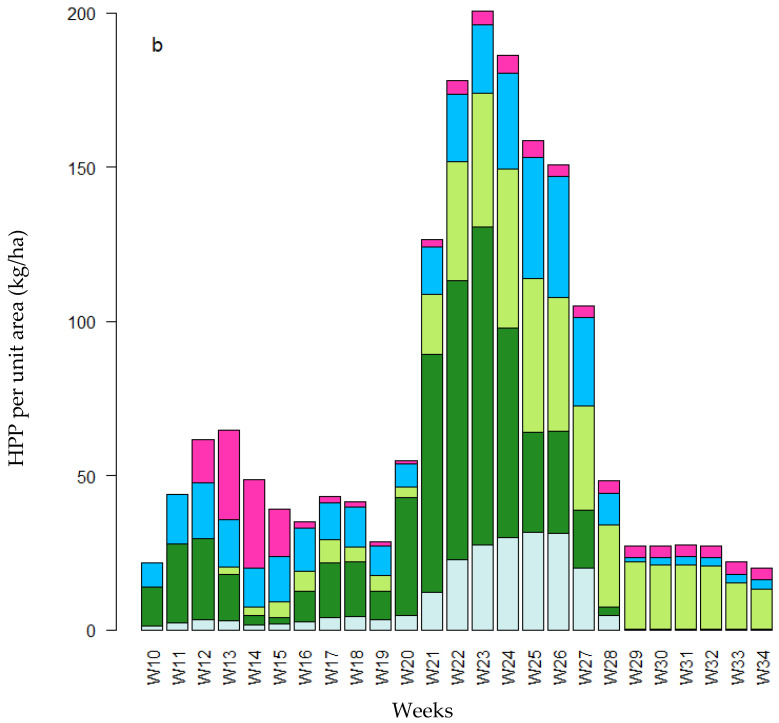
Temporal variation in cumulative HPP per week as a function of land use type: (**a**) total HPP (×10^8^ kg); (**b**) HPP per unit area (kg/ha).

Figure 4 provided a clear picture of the temporal variability and the general impact of each type of land cover considered. Four periods followed one another in the honey production potential between weeks 10 and 34 (Figure 4a). The first concerned weeks were 10 to 15. It showed rather low levels of HPP with a major contribution from the forests. However, in the downward phase, the orchards made a significant contribution to the weekly HPP in weeks 12 to 15, with, for example, up to 45% of the HPP in week 14 coming from the orchards. Weeks 14 and 15 showed the lowest weekly HPP and similar values were not seen again until August. The second period ran from weeks 16 to 19 and was characterized by a greater contribution from grassland, up to 21.5% in week 17. The contribution from orchards became negligible from week 16 onwards. The third period ran from week 20 to 27. It was characterized by an increase in the contribution from all the LUSs, which contributed to a very significant increase in the weekly HPP, with a maximum value in week 23 for the season. From weeks 20 to 26, the forest made the largest contribution then equaled the contribution from grassland in weeks 25, 26, and 27. Weeks 21 to 27 allowed a contribution from wasteland, up to 10% in week 25. In the fourth period, from week 28 and almost all in weeks 29 to 34, grassland accounted for most of the HPP. Although forests represented the largest share of potential honey production, meadows provided a steady and substantial foraging resource throughout June, July, and August. This indicated the importance of grassland as a significant foraging area for bees. The contribution of riparian vegetation remained very low, with an increase between weeks 21 and 27.

The importance of forest and grassland in the establishment of total HPP was clearly visible. Nonetheless, rarer land used such as orchards, riparian forests, and wasteland were useful insofar as they represented a significant contribution to the HPP in relation to their surface area (Figure 4b). Using the periods described above, their relative involvement varied greatly. In the first phase, the involvement of orchards and riparian vegetation was very high, reaching 59.3% and 25.7% respectively in week 14, while grassland and forest were at 5.31% and 6.20%, respectively. In the second phase, forest and riparian forest shared the bulk of the contributions, as in week 18, when they accounted for 42.6% and 31.2% respectively. The third phase saw the most significant implications for all land uses except orchards. Initially dominated by forest, the implications were equivalent in weeks 25, 26, and 27. Finally, in the last phase, grassland dominated the implications in relation to the other land uses.

### 3.5. Contribution of the Main Taxa to Overall HPP

**Figure 5:**
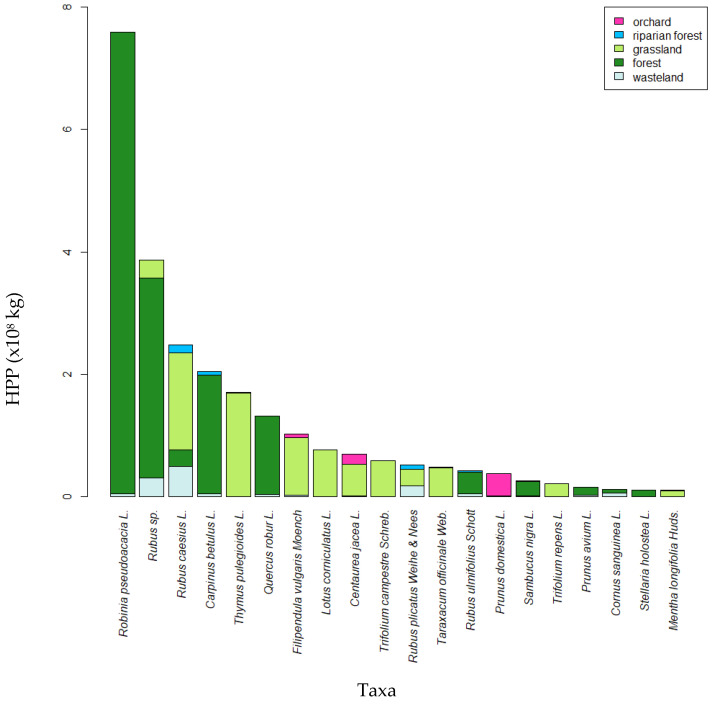
Cumulative HPP over 25 weeks of the 20 taxa contributed most to the total HPP of the foraging area.

Identifying the contribution of each taxon to the overall HPP by considering all the observation points enabled to characterize the most abundant taxa (Figure 5). These were mainly found in forests, with trees such as *Carpinus betulus* or *Robinia pseudoacacia*, but also *Rubus*. Grasslands also played an important role, as ten of these major taxa were mainly found in this LUS. Forests and grasslands were the main hosts for these taxa, but land-use types less important in terms of surface area were heavily involved for certain abundant taxa, where, for example, 24.7% of the surface area covered by *Rubus caesius*, 48.8% for *Rubus plicatus* and up to 55.8% for *Cornus sanguinea* were attributed to wasteland and riparian vegetation. Similarly, in the case of orchards, *Filipendula vulgaris* accounted for 6.02%, *Centaurea jacea* for 23.3%, and up to 96.9% for *Prunus domestica*, the main species cultivated in these orchards.

## 4. Discussion

The diversity of melliferous resources within a landscape makes it possible to supply the colony with complementary nutritional inputs that maximize its fitness [57]. A variety of melliferous resources which will follow one another in time due to their specific phenology, and in space due to the diversity of communities associated with land use, will make it possible to offer colonies resources in sufficient quantity and quality to meet the diverse of their physiological needs. As *Apis mellifera* is a polylectic pollinator [58] and the colony is structured as a complex system [59], the organization of foraging makes it possible to take advantage of the different spatial and temporal opportunities for access to melliferous resources [60,61,62]. Wasteland and riparian zones are particularly interesting because they combine several vegetation strata, often in a small area, making available a diversified melliferous resource [63] and offering staggered flowering throughout the season [54]. On the other hand, landscapes characterized by a large number of arable plots have a strong impact on bee colony dynamics [64]. In our example, the presence of arable plots did not provide colonies with melliferous resources, apart from maize, which provides a potential pollen resource. Nevertheless, the small surface area allocated (less than 10% of the foraging area considered) remained small compared with other types of LUS and the impact on colony dynamics hence remained small. Similarly, the HPP responded to a question about honey production from nectar resources, which excluded maize. Studies carried out in landscapes with a high proportion of arable crops [62,65] had identified a harvest peak in April followed by a trough in June. In the case of our study, April was a low period, partly offset by the flowering of orchard plots, and June corresponded to the highest potential with the transition in exploitable melliferous resources from forests to meadows. Several studies [10,16,66] have shown an increase in flight distances due to the scarcity of melliferous resources during the summer months, particularly in July. Our study is in line with this trend, showing a decrease in HPP in summer, with resources almost exclusively provided by meadows. Similarly, [16] showed that the ‘summer blues’ are more pronounced in forested landscapes than in more open environments. Our study, which shows a forest landscape at less than 40%, shows a 5.7-fold decrease in overall HPP between periods 3, which relied mainly on forest resources, and 4, which relied on grassland resources.

The number of species potentially exploitable by bees within the foraging area (162 species) is consistent with other results of pollen analysis studies (between 149 and 168 species, in particular for [67] which worked on the subspecies *Apis mellifera carnica* present in the Balkans). Four of the twenty most important plant species contributing to the overall HPP for our study are found in [55], nine if we generalize to genera, for a study area located around 200 km away as the crow flies. Most of the HPP identified in the foraging area is borne by a small number of plant species. However, it has been shown that the diversity of the resources visited, as well as their sufficient availability throughout the colony’s activity, has a strong impact on the colony’s long-term survival [11]. This diversity will make it possible to meet the colony’s nutritional needs and improve the general health of the individuals making up the colony [68]. Moreover, landscape diversity does not influence the abundance and diversity of the resources collected. Low values in the nearby landscape are compensated for by the exploitation of a larger foraging area [11]. This leads to a greater flight distance per forager in simple landscapes compared with more complex ones [14]. Wasteland could be interesting to increase the diversity of potential melliferous resources. This LUS was characterized by several strata (shrub, bush, sometimes with a herbaceous stratum) made up of spontaneous vegetation, formerly maintained and/or cultivated then abandoned and evolving towards a forest formation [69]. Several species are abundant there, especially *Rubus* sp., even if, in our study, the total plant species was lower than other LUSs, and weeks with the highest values of HPP contribution coincide with forest and grassland ones.

This work enabled us to model the theoretical availability of the melliferous resource for bees by identifying the most attractive sectors in space and time. However, this upstream availability does not condition the actual ability of bees to visit flowers and bring nectar and pollen back to the hive. In [70], only half of the available melliferous resources were found in honey and beebread. In natural habitats, the proportion of flowering species not utilized in colony products can reach up to 60%, depending on the time of year. Estimating the nectariferous potential of each taxon poses a challenge. Nectar production depends on the plant’s physiology. The quantity of nectar produced and available fluctuates during the day, via hormonal control, particularly by jasmonate [71], but also by competition for food with other pollinators [72]. Nectar produced but not used by a pollinator may even be reabsorbed by the plant [73]. Experimental methods can be used to monitor fluctuations in production and availability, in particular by collecting nectar inside the flowers [74,75], but they are difficult to set up and monitor. Modelling can also be used to monitor nectar potential [74,76]. Finally, remote sensing approaches have been proposed to anticipate and assess flowering, some of them even proposing an evaluation of honey production [77,78,79,80]. However, although these approaches are useful for obtaining a general view of the production potential over a large area with homogeneous resources, such as in the context of large plots, large orchards, or little diversified forests, it is difficult to use in the context of specific work on the capacity of an apiary to exploit a restricted area such as a foraging area, in a heterogeneous landscape such as northern B&H.

The ability of a colony to exploit a foraging area and its resources depends on a variety of factors: organoleptic and chemical characteristics of the products harvested from the resource [81,82,83], ecological characteristics of the populations and communities of the plants of interest, and structure of the landscape [84], phenology and capacity to supply nectar and/or pollen [85], individual and collective ethological characteristics of bees (distance to the hive, resource encountered by a scout, recruitment within the hive) [22,86], weather conditions which will influence the phenology of the plant and the capacity of the foragers to leave the hive and ensure the harvest [87,88,89]. Our model focused on the capacity of the resource to produce and the interaction with a colony but did not take into account the dynamics of the colony and its capacity to forage effectively. Models of interaction with the climate and colony dynamics [76,90,91,92] could be coupled to our approach in order to model more finely the effective interaction between a bee colony and a melliferous resource in order to come closer to a coherent prediction of the productivity of an apiary. Similarly, pollinator communities within ecosystems coexist and interact to exploit resources. A detailed understanding of an area’s capacity to provide effective honey production therefore requires the integration of the effect of other pollinators. Pollinator-resource networks are complex and specific [72,93] and contribute to the ecosystem. Honeybees integrate these networks through direct and indirect competition [94]. Competition between different apiaries also remains problematic insofar as the functioning of colonies remains similar. Several studies have attempted to optimize the positioning of apiaries according to the potential productivity of a zone [77,95,96]. One of the possible perspectives of our study could be the generalization of the understanding of the space-time variation of HPP over a zone larger than the foraging area in order to optimize the positioning of apiaries and minimize mutual competition.

## 5. Conclusions

This study is part of the drive to better understand the interaction between bees and the environment. In particular, it enabled us to map the spatial and temporal variations in HPP in an area close to an apiary, which is most used by the colonies. It made it possible to associate the specific features of the landscape, particularly in terms of land use, with the production capacity of an apiary. Successions over time in terms of the contribution made by LUS, as well as the intensity of the production potential, could be highlighted in detail, down to the week. It was also possible to identify the plant species that make the greatest contribution to HPP. The results presented in this study are largely in line with the literature and reflect the specific features of a contrasting landscape environment where forest dominates in the establishment of HPP. This work is an example of a detailed understanding of the environment around the apiary, corresponding to the questions beekeepers are asking about their ability to anticipate the production potential of an area, both in terms of the reasoning behind honey flows and the triggering of transhumance.

## Figures and Tables

**Table 1:** Mean monthly, maximum, and minimum temperatures for 2022 [28], 2023 [29], and 1991–2020 period [30].

	Month	Jan.	Feb.	Mar.	Apr.	May	June	Jul.	Aug.	Sept.	Oct.	Nov.	Dec.
Temperature (°C)	Mean monthly in 2022	0.60	5.00	4.90	10.3	17.8	22.7	22.4	21.9	16.3	13.5	7.70	5.30
	Mean monthly in 2023	3.80	3.20	8.70	10.3	15.6	20.2	22.7	21.6	18.8	15.6	8.40	4.90
	1991–2020 mean	0.64	1.95	5.73	10.2	14.7	18.4	20.4	20.6	15.9	11.0	6.03	1.09
	Mean monthly minimum in 2022	−4.00	−0.60	−2.00	3.70	10.8	15.2	14.6	15.8	11.0	8.50	4.20	2.10
	Mean monthly minimum in 2023	0.30	−2.30	2.40	5.00	11.4	14.4	16.4	15.4	12.7	9.80	3.30	0.40
	Mean 1991–2020 minimum	−2.70	−1.97	0.94	5.10	9.23	12.8	14.3	14.4	10.1	6.09	2.26	−2.03
	Mean monthly maximum in 2022	6.30	12.6	13.1	17.6	25.2	29.8	30.6	28.9	23.8	21.4	12.3	9.20
	Mean monthly maximum in 2023	8.40	10.0	16.1	16.2	20.8	26.6	30.4	29.3	26.8	23.4	14.0	10.9
	Mean 1991–2020 maximum	3.98	5.89	10.6	15.4	20.2	23.9	26.6	26.9	21.8	16.0	9.84	4.21

Data for 2022 and 2023 were measured at the Sanski Most station, 30 km from the study site, and data for the period 1991–2020 were assessed for the Republic of Srpska using data compiled by the Climatic Research Unit of the University of East Anglia.

**Table 2:** Landscape characterization of the type of land use.

Type of Land Use	Number of LUUs	$S_{i}$ (ha)	${Dt}_{i}$ (LUU/100 ha)	$ENN_{i}$ (m)
Cropfield excluding corn	43	0.685 ± 0.52 ^ab^	6.08	118 ± 110 ^a^
Forest	40	6.41 ± 11.0 ^a^	5.66	51.4 ± 51.7 ^bc^
Wasteland	38	1.68 ± 2.95 ^a^	5.38	81.9 ± 112 ^abcd^
Maize	49	0.583 ± 0.50 ^b^	6.93	99.7 ± 106 ^ab^
Grassland	114	1.62 ± 3.44 ^ab^	16.1	24.7 ± 28.7 ^d^
Riparian forest	13	0.929 ± 1.28 ^abc^	1.84	72.3 ± 142 ^abcd^
Orchard	132	0.327 ± 0.545 ^c^	18.7	29.5 ± 45.5 ^cd^
Statistical analysis	-	Χ^2^ = 116.84, df = 6, *p* < 0.001	-	Χ^2^ = 71.35, df = 6, *p* < 0.001

Values are given in mean ± standard deviation. Different superscript letters within a column indicate significant differences (two-by-two multiple comparison test).

**Table 3:** Total and average plant richness of the research area.

Land Use Type	Total Plant Species	Average Plant Species Number per Observation Plot
Forest	66	5.39 ± 3.07 ^a^
Wasteland	50	5.78 ± 2.65 ^a^
Grassland	84	14.4 ± 6.05 ^b^
Riparian forest	69	16.7 ± 5.88 ^b^
Orchard	45	-
Statistical analysis	-	Χ^2^ = 59.12, df = 3, *p* < 0.001

For the statistical analysis, values are given in mean ± standard deviation. Different superscript letters within a column indicate significant differences (two-by-two multiple comparison test).

## Data Availability

Data are available upon request from the authors.

## Abbreviations

The following abbreviations are used in this manuscript:

B&H	Bosnia & Herzegovina
GIS	Geographical Information System
HPP	Honey Production Potential
LUS	Land Use Station
LUU	Land Use Unit
NDVI	Normalized Difference Vegetation Index
